# Mild Disease Course of Experimental Autoimmune Encephalomyelitis without Pertussis Toxin: Brain Transcriptome Analysis Reveals Similar Signaling to Active Lesions in Multiple Sclerosis

**DOI:** 10.3390/biomedicines12061215

**Published:** 2024-05-30

**Authors:** Christa M. Frodella, Stephen B. Pruett, Barbara L. F. Kaplan

**Affiliations:** 1 Department of Comparative Biomedical Sciences, College of Veterinary Medicine, Mississippi State University, Starkville, MS 39762, USA; christa.frodella@gmail.com (C.M.F.); pruett@cvm.msstate.edu (S.B.P.); 2 Center for Environmental Health Sciences, Department of Comparative Biomedical Sciences, College of Veterinary Medicine, Mississippi State University, Starkville, MS 39762, USA

**Keywords:** multiple sclerosis, experimental autoimmune encephalomyelitis, neuroinflammation, G protein coupled receptors

## Abstract

Experimental autoimmune encephalomyelitis (EAE) is a powerful model to study multiple sclerosis (MS). One of the approaches for EAE is to actively immunize with myelin-derived peptides with immune adjuvants. One of the commonly used immune adjuvants is pertussis toxin (PTx), without which EAE disease is mild with relatively longer onset. However, pertussis toxin can also inhibit G protein-coupled receptor (GPCR) signaling so it can confound investigations into the role of GPCRs in EAE or therapies designed to target GPCRs. Since EAE via active immunization without PTx results in a relatively mild disease state, we wanted to confirm that appropriate signaling molecules for the disease were being induced in one target tissue (i.e., brain). RNA-Seq analysis of whole brain tissue demonstrated that the MS signaling pathway was strongly activated in symptomatic mice. In addition, there was activation of Th1 (IFN signaling), Th2 (IL-4 signaling), and Th17 (IL-17 signaling). In comparing canonical pathways from our mouse mild EAE brains with a human MS atlas, EAE shared the most pathways with active and inactive lesions. An advantage of this approach is that disease induction is slower to develop and results in modest clinical signs, which likely more closely mimic human disease onset.

## 1. Introduction

Experimental autoimmune encephalomyelitis (EAE) is an invaluable animal protocol to study human multiple sclerosis (MS). Both EAE and MS are immune-mediated diseases that cause neuroinflammation and demyelination of the central nervous system (CNS) [1]. Through EAE research, several therapeutics such as glatiramer acetate [2] and mitoxantrone [3] have been translated into the clinic to treat MS. However, not all therapeutics have been successful in the bench-to-clinic transition. For example, blockade of tumor-necrosis factor (TNF) receptor improved EAE but worsened MS [4]. Given this bench-to-clinic challenge, refining the EAE model might provide additional insights for pre-clinical testing.

Currently, a popular approach for the EAE model is active EAE in which a protein component of myelin is injected with an immune adjuvant to initiate an immune response against cells that express myelin. Specifically, an emulsion is injected subcutaneously that contains a peptide fragment of myelin oligodendrocyte glycoprotein (MOG), Complete Freund’s Adjuvant (CFA) comprised of mineral oil, and sometimes supplemented with heat-killed Mycobacterium tuberculosis H37Ra (HKMT). In this model, especially in C57BL/6 mice, pertussis toxin (PTx) is also injected intraperitoneally [1]. Notably, PTx enhances immune cell entry into the CNS [5]. However, despite the disease-inducing efficacy of PTx, it does inactivate G-inhibitory protein-coupled receptors [6]. Therefore, PTx might confound experiments assessing therapeutics that act through G protein-coupled receptors (GPCRs), including cannabinoids [7,8].

Cannabidiol (CBD) is one example of a putative cannabinoid therapy for MS that might act through GPCRs. CBD is a phytocannabinoid found in *Cannabis* sp. that has structural similarity to Δ9-tetrahydrocannabinol (THC) but lacks the adverse cognitive side effects associated with THC [9]. CBD has been shown to exhibit efficacy in various EAE models [10,11,12,13,14,15,16,17]. CBD has also shown promise in MS when used in combination with THC as the oromucosal spray therapeutic (Nabiximols) for treatment of spasticity [18,19,20]. However, CBD’s mechanism of action is not fully understood and might work through GPCRs, such as cannabinoid receptors (CB_1_ and CB_2_), G protein-coupled receptor 55 (GPR55), or adenosine receptors [21]. Therefore, to further test the therapeutic efficacy of CBD or other putative therapeutics that might act through GPCRs, it was first necessary to investigate the degree to which active EAE could be induced without PTx as a mild model of EAE.

Previous research in our lab has shown that without PTx, our mild EAE model is effective and results in clinical presentations (i.e., tail and hind limb paralysis), albeit the symptoms are milder, variable (i.e., not all mice develop symptoms), and with more prolonged onset [16,22,23,24,25,26]. These facets of our mild EAE model offer an area of interest to analyze the variability in disease that may mimic mild MS in humans. To further elucidate the mechanisms that drive our mild EAE model, mild EAE symptom variability, and how these mechanisms compare to human MS, an RNA sequencing (RNA-Seq) analysis was performed on symptomatic and asymptomatic mild murine EAE brains and compared to transcriptomic pathways in various post-mortem MS brain lesions. Thus, the specific research objectives of this study were to identify signaling pathways in the brains of mice that developed EAE using our modest induction model, compare signaling pathways in EAE mice in which clinical disease was observed to those in which clinical disease did not develop, and compare brain signaling pathways in our EAE mice with post-mortem brains from individuals with MS. We hypothesized that signaling in the brain would be consistent with EAE and MS using active immunization with MOG_35–55_ in CFA without PTx in C57BL/6 mice. This study is novel because we will establish a transcriptomic profile for clinical disease take using active immunization without PTx and classify our model in the context of human MS. Results from this study provide justification for this approach as a mild model of EAE that mimic mild MS in humans.

## 2. Materials and Methods

### 2.1. Reagents

MOG_35–55_ peptide (MEVGWYRSPFSRVVHLYRNGK) was obtained from Biosynthesis (Lewisville, TX, USA). HKMT was purchased from Difco/BD Biosciences (Detroit, MI, USA). CFA was obtained from Sigma (St. Louis, MO, USA). In addition, the following primers were purchased from ThermoFisher (Waltham, MA, USA) for quantitative real-time reverse transcription polymerase chain reaction (qRT-PCR): *Tnfa* (Mm00443258_m1) and *Ifng* (Mm01168134_m1).

### 2.2. Animals

Female C57BL/6J mice were purchased from Envigo (Indianapolis, IN, USA). Five mice were housed per cage and allowed to drink and eat ad libitum. Cages were maintained at a temperature of 22 ± 1 °C, 40–60% humidity, and a 12-hr-light:12hr-dark-controlled room. EAE was induced in adult mice (10 weeks). Since EAE causes paralysis, food and water access were ensured by placing food pellets on the floor and using longer sipper tubes as the disease progressed. Protocols were performed in an AAALAC-approved facility per Mississippi State University Institutional Animal Care and Use Committee (protocol 19-273 to BLFK).

### 2.3. Induction and Assessment of EAE

Mice were anesthetized using 3% isoflurane and immunized with CFA containing the MOG_35–55_ peptide and HKMT. Each mouse was injected subcutaneously over the shoulders and hips with 100 µL of CFA containing 500 µg of HKMT and 100 µg of MOG_35–55_ peptide. Control mice received 100 µL saline. Injections were divided over four injection sites using 25 µL/site. Approximately 24 h after EAE disease induction, mice were dosed with 100 µL corn oil (CO) via oral gavage over five days. Although no therapies were evaluated in this study, we included CO as it does serve as the vehicle for various treatments that we intend to assess in this model. Mice were then observed for 18 days, and clinical scores were given based on the following scale as described [17]: 0—Asymptomatic; 0.5—Flaccid tail; 1—Hindlimb paresis/waddling; 1.5—Waddling gait; 2—Unable to prevent being placed in dorsal recumbency; 2.5—Hindlimb dragging; 3—Single hindlimb paralysis; 3.5—Single hindlimb paralysis with other hindlimb dragging; and 4—Complete hindlimb paralysis. Mice were not allowed to advance past a score of 4 to ensure animal welfare. In total, the scores and samples analyzed were from two independent cohorts of three mice per group in each cohort (designated as A and B). Mice were divided into two treatment groups: control (saline/CO) or EAE (EAE/CO). EAE/CO mice that developed clinical disease were deemed symptomatic, and EAE mice that received a score of zero were considered asymptomatic. Control mice did not exhibit clinical disease and were not used in further analyses.

On day 18 (D18), brains were flash-frozen in liquid nitrogen and stored in RNA, later at −80 °C. RNA isolation, RNA quality evaluation, cDNA library construction, and Illumina RNA sequencing (RNA-Seq) from EAE brains were performed by NovoGene (Sacramento, CA, USA). Reads were paired-end, 150 base pairs at a depth of 40 million reads. Reads were assessed for quality, trimmed, and mapped to the GRCm39 genome (NCBI), and differential expression was analyzed using CLC Genomics Workbench (Qiagen, Germantown, MD, USA) using default parameters. Differentially expressed genes (DEGs) with a false discovery rate (FDR) ≤ 0.05 were considered significant. The CLC Differential Expression RNA-Seq workflow utilizes a multi-factorial statistical model based on a negative binomial Generalized Linear Model (GLM) to identify statistically significant genes between groups.

### 2.4. MS and Control Brain Tissue Transcriptomic Analysis

An MS atlas was downloaded from the gene expression omnibus (GEO) database (GSE138614) [27]. Control white matter from healthy subjects and tissue in various stages of progressive MS were RNA sequenced. The following post-mortem samples were analyzed: (1) active lesions, (2) chronic active lesions, (3) inactive lesions, and (4) remyelinating lesions. Reads were assessed for quality, trimmed, and mapped to the GRCh38 genome (NCBI), differential expression analysis (each lesion compared to control white) in CLC Genomics Workbench (Qiagen). Differentially expressed genes (DEGs) with an FDR ≤ 0.05 and fold-change ≥ 1.5 were considered significant.

### 2.5. Ingenuity Pathway Analysis

DEGs from EAE symptomatic and MS lesions were analyzed with Ingenuity Pathway Analysis (IPA, Qiagen). Canonical pathways were deemed significant with a −log (*p*-value) ≥ 1.3 (equal to *p*-value ≤ 0.05) and a |z-score| ≥ 2. Negative and positive z-scores indicated that the pathways reflected inhibition and activation, respectively.

### 2.6. RNA Extraction and RT-qPCR

Selected pro-inflammatory genes, *Tnfa* and *Ifng*, were validated by real-time quantitative polymerase chain reaction (RT-qPCR) in EAE brains. Total RNA that we received back from NovoGene was reversed transcribed using random primers with the High-Capacity cDNA Reverse Transcription Kit (Applied Biosystems/ThermoFisher, Waltham, MA, USA). cDNA was amplified with Taqman primers and probe sets and analyzed using a Real-Time PCR System Agilent Stratagene Mx3005P. Fold-change values were calculated using the ΔΔCt method with the internal reference 18s rRNA and EAE/CO Symptomatic as the comparator [28].

### 2.7. Statistics

Statistical analyses were performed using GraphPad Prism version 7 (San Diego, CA, USA). The mean ± standard error mean (SEM) was determined for fold-changes for each gene analyzed, and RT-qPCR fold-changes were transformed using a natural log (fold-change + 1) prior to ANOVA analysis. A two-way ANOVA was performed, and a *p*-value < 0.05 was deemed significant.

## 3. Results

### 3.1. Mild EAE Clinical Scores Exhibit Variability

In our study, the mild EAE disease induced in the absence of PTx typically peaked between D18-24 [17,22,23,24,25,26]. Therefore, clinical scores were assessed from D13-18, and mice were euthanized on D18. Symptomatic mice presented with scores that ranged from 0 < score ≤ 4, and asymptomatic mice were assigned a score of zero. Since this EAE disease model is mild, not all mice developed symptoms as defined (50% incidence). As such, select mice from each cohort (designated A and B) were chosen to determine the transcriptomic mechanisms of mice that developed EAE (i.e., symptomatic). Brains from symptomatic mice (*n* = 3) with the highest symptomatic scores and select asymptomatic mice with zero scores (*n* = 3) were used for RNA-Seq analysis. Symptomatic mice exhibited an average score of 2.3 over the three mice (Table 1).

### 3.2. EAE Mice Present with Numerous DEGs and Immune-Related Canonical Pathways

**Figure 1:**
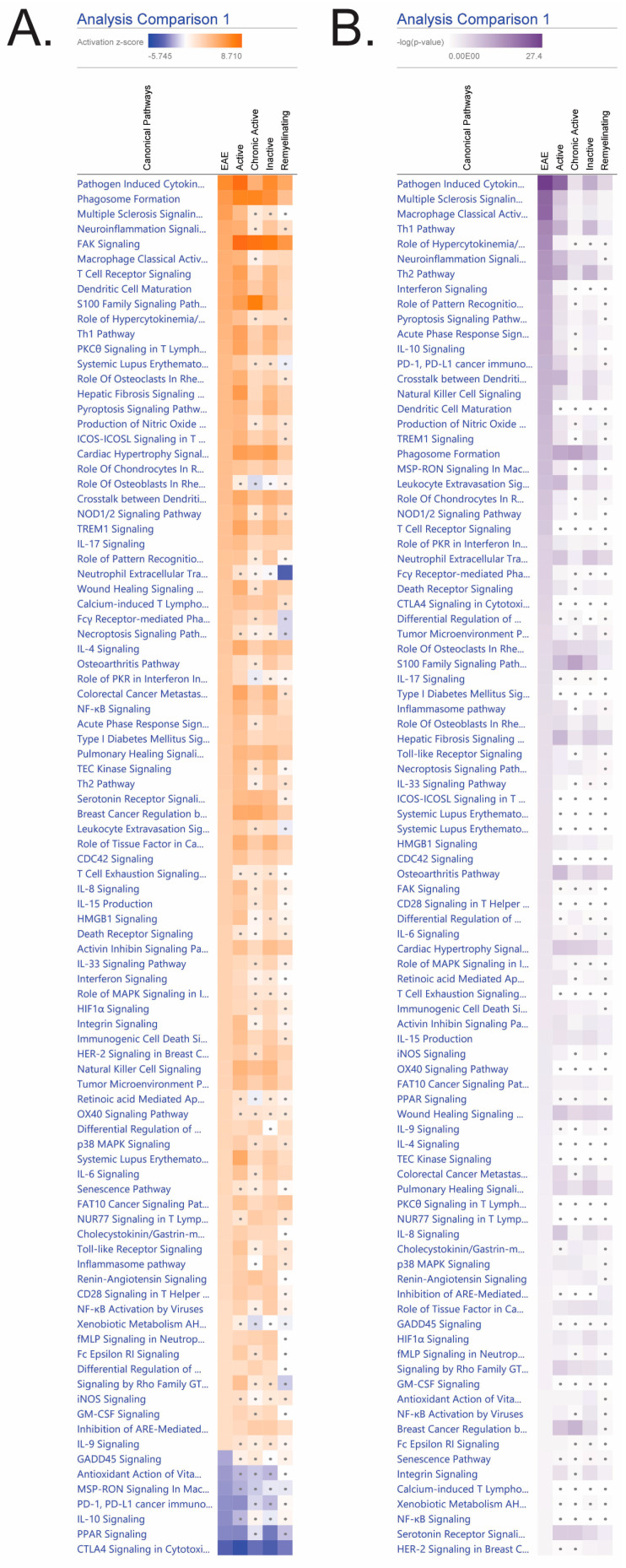
Symptomatic EAE mouse brains and MS lesions share numerous canonical pathways. All EAE symptomatic brain pathways that were significant for (**A**) activation |z-score| ≥ 2 and (**B**) −log (*p*-values) ≥ 1.3 (i.e., *p*-value < 0.05) that were listed here and analyzed for comparison against MS lesions (active, chronic active, inactive, and remyelinating). For differential expression, all lesions were compared to control white matter. Inflammation, innate and adaptive immune, cytokines, cell mobility, tissue damage, and anti-inflammatory pathways and signaling were included. Activated and inhibited pathways (z-score) exhibited orange or blue gradient colors, respectively. For −log (*p*-values) are demonstrated in purple gradients. Darker pathways correlate with a more significant pathway. Pathways that were non-significant in activation/inhibition and −log (*p*-values) were denoted with gray dots.

In EAE symptomatic mice, there were 354 DEGs, with 345 and nine genes that were increased and decreased in the brains, respectively. The significant canonical pathways were analyzed using IPA (Qiagen) for each comparison. Canonical pathways were deemed significant with a −log (*p*-value) ≥ 1.3 (equal to *p*-value ≤ 0.05) and a |z-score| ≥ 2. Negative and positive z-scores indicated that the pathways reflected inhibition and activation, respectively. EAE symptomatic mice exhibited 92 canonical pathways with 85 activated and seven inhibited pathways in the brains. The top 20 statistically significant canonical pathways are presented (Table 2); notably, the MS signaling pathway was strongly activated (Figure 1). Additionally, the immune phenotype appears to be heterogenic. Presented in Table 2 is a strong activation of the Th1 and Th2 Pathways; Table 3 shows a mix of Th1-(IFN signaling), Th2-(IL-4 signaling), and Th17-(IL-17 signaling) associated cytokines [29].

### 3.3. Mild EAE Brain Presents Share Numerous Canonical Pathways with MS Lesions

**Figure 2:**
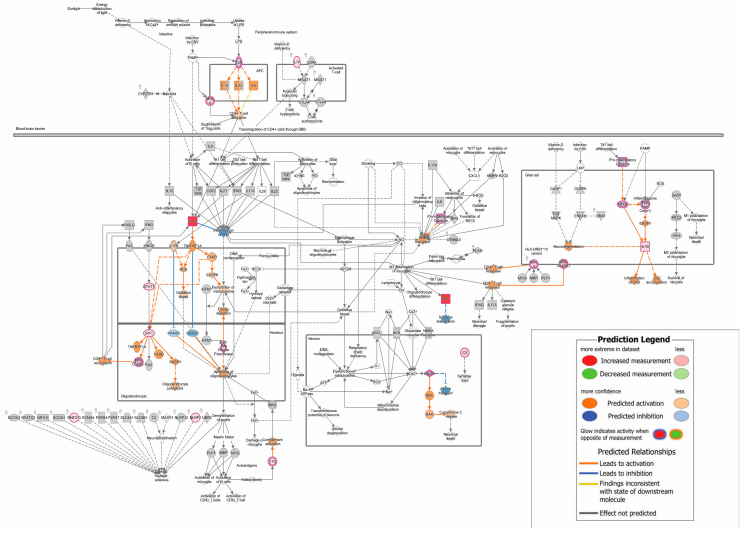
MS signaling is activated in EAE symptomatic mouse brains. Dataset molecules are highlighted in pink and double borders are indicative of a group or complex. The colors red and green reflect measured increased upregulation and downregulation, respectively. Additionally, molecules highlighted in orange and blue reflect predicted activation and inhibition, respectively. A gradient of colors signals a group or complex of molecules with disparate expression. Grey nodes indicate the molecule was present in the dataset but did not pass defined filters. White nodes are molecules that are not present in the dataset.

Considering that the EAE model utilized in this experiment is mild, it was crucial to assess the pathways that are a part of disease progression (e.g., innate and adaptive immune mechanisms) and how the model compares to transcriptomic pathways of known MS lesions. EAE mice exhibited an overall activation in inflammatory pathways (e.g., pathogen-induced cytokine storm, neuroinflammation, and acute phase response) and inhibition in anti-inflammatory pathways (i.e., IL-10 and PPAR). Significant pathways (*p* ≤ 0.05 and |z| ≥ 2) in EAE analysis were compared to activation and inhibition gradients in MS lesions (active, chronic active, inactive, and remyelinating). To contain analysis in reference to EAE, pathways that were significant in MS lesions but not EAE were not listed. Therefore, only pathways that were significant in EAE (i.e., *p*-value ≥ 1.3 and |z-score| ≥ 2) were provided and used to compare to MS lesions. Non-significant pathways were denoted with gray dots. There were 92 significant and activated/inactivated pathways for EAE symptomatic mice (Figure 2). EAE shared the most pathways with active (76/92 pathways) and inactive lesions (71/92 pathways). EAE brains were least comparable to chronic active (43/92 pathways) and remyelinating (37/92) lesions.

### 3.4. Neuroinflammatory Genes Expression Increased in EAE Symptomatic Mouse Brains

**Figure 3:**
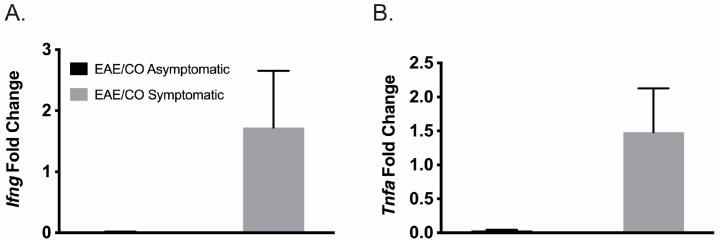
EAE neuroinflammatory gene expression of *Tnfa* and *Ifng* in EAE. Neuroinflammatory gene expression of pro-inflammatory cytokines *Tnfa* (**A**) and *Ifng* (**B**) in EAE groups are presented as mean ± SEM. Symptomatic EAE values were used as the fold-change analysis comparators.

Two major neuroinflammatory cytokines, *Tnfa* and *Ifng*, were analyzed via RT-qPCR to confirm the inflammatory state of EAE. As evidenced in Figure 3, *Tnfa* and *Ifng* expression were increased in the brains of symptomatic EAE mice compared to the asymptomatic groups, which is consistent with increased clinical scores and activated inflammatory canonical pathways.

## 4. Discussion

The overarching goal of this experiment was to elucidate the transcriptomic mechanisms that drive a mild model of EAE (i.e., active immunization using MOG_35–55_ without PTx) and determine its robustness as a model to study MS for therapeutics that might require functional GPCRs. The clinical presentations in our model are variable, modest, and slower to develop compared to the EAE models that include PTx [17,22,23,24,25,26]. However, this model likely mirrors mild MS progression in humans as comparisons between mild EAE and MS canonical pathways showed similarities with the active MS lesions. It is also worth noting that the presence of lesions does not always lead to an impaired neurological system and can be classified as “clinically silent multiple sclerosis lesions” [30]. Indeed, previous research in our lab has shown that clinically asymptomatic EAE mice still exhibited inflammation in their spinal cords [31].

There are other EAE models that don’t require PTx and could therefore also be used to evaluate the role of GPCRs and/or potential therapies that act via GPCRs without the potential confound of GPCR inhibition by PTx. For instance, active immunization with another protein called proteolipid protein (PLP) does not require PTx for induction of reliable EAE [32]. In addition, typically, passive EAE in which encephalitogenic T cells are adoptively transferred to healthy recipient mice will induce EAE without PTx [32]. These two models have the advantage that they produce more robust disease in a shorter time frame as compared to active EAE using MOG_35–55_ peptide immunization without PTx as shown here. One limitation with the passive EAE model, however, is the dependence solely on T cells to transfer disease. We have established in our mild model that we can induce MOG_35–55_-specific IgG [23] and MOG_35–55_-specific IgG1 [25] in addition to MOG_35–55_-specific T cell responses, which is another advantage of our mild EAE model.

GPCR involvement in EAE is clear from the fact that most chemokine receptors are Gi GPCRs [33] and have been suggested as druggable targets for MS [34]. Other studies using GPCR knockouts have provided evidence that GPCRs contribute to either the pathophysiology or regulation of EAE disease. For instance, deletion of the orphan GPCR GP141 (*Gpr141^−/−^* mice) exacerbated EAE [35]. Similarly, we noted exacerbated EAE disease in cannabinoid receptor 1 (CB_1_) knockout mice (*Cnr1^−/−^)* [31]. It might be expected that knockout of a Gi/o GPCR gene would regulate EAE in a similar manner to use of PTx since both would result in inactivation of the GPCR, although it should be noted that PTx treatment likely inactivates more than just one GPCR but is not a permanent inactivation. That inactivation of a Gi/o GPCR exacerbated EAE also suggests that targeting these receptors might be therapeutic. Indeed, it has been shown that CB_1_ expression on neurons was required for cannabinoid-mediated suppression of EAE [36].

A limitation of our mild EAE model is a relative lack of dependence on IL-17 as compared to more robust EAE models that use PTx. Indeed, as shown here, the Th1 pathway and IFN signaling were more significantly induced as compared to Th17 pathway or IL-17 signaling. In previous findings with this this model, we have also seen more robust upregulation of IFN-γ as compared to IL-17 in MOG_35–55_-restimulated splenocytes or serum [16,26,31]. There is at least one other EAE model in which IL-17 played a minor role; MOG plus IL-23-stimulated encephalitogenic T cells were able to transfer EAE disease to *Il17^−/−^* recipient mice in an IFN-γ-dependent manner [37], so the relative lack of dependence on IL-17 is more broadly applicable to other cell types involved in EAE. Other limitations of our study are the small number of animals analyzed, and that we conducted the analysis in whole brain tissue so we cannot identify the cell type(s) that were mediating the cytokine production or signaling. For instance, it has recently been identified that there exists a dual role for IFN-γ on microglial cells in EAE with relatively high IFN-γ promoting inflammation and lower IFN-γ providing neuroprotection [38]. However, we cannot determine from this work the source(s) of IFN-γ, nor the degree to which various cell types might be responding to IFN-γ, in the brains.

Despite the relatively lower involvement of IL-17 in our mild EAE model, it did exhibit similar signaling and canonical pathways to active and inactive MS lesions. The high degree of active lesion similarity was expected as active lesions are associated with demyelination [39,40] and typically present with clinical symptoms [41]. However, inactive lesions correspond to residual inflammation with no active demyelination [42] and might reflect mild inflammation in the EAE brains. It was expected that signaling in our mild EAE model would not mimic chronic active MS lesions or remyelinating lesions. Chronic active lesions are associated with the severe nature of progressive MS and shorter time to disability presentations [43], which is not a characteristic of a mild MS model. Lastly, the remyelinating lesions were not anticipated as mice were euthanized 18 days past disease induction, which does not allow much time for a remyelination phase, nor does active immunization with MOG_35–55_ in C57BL/6 typically induce a relapsing-remitting model of MS [32].

Although the mice in this study were grouped for RNA Seq analysis based on their gross clinical scores a priori (i.e., symptomatic versus asymptomatic), we have considered the possibility that the currently accepted assessment of clinical scoring for EAE might not best reflect our mild EAE model. As we have stated, we have noted that neuroinflammation can be detected in the spinal cords in mice that have received a clinical score of zero [31]. However, as compared to a simple gross clinical assessment, histological assessment of spinal cord inflammation is quite invasive, time-consuming, and can only be determined at study’s end. Perhaps in our hands, quantification of the ratio of IFN-γ to IL-17 in the serum might be valuable as this can be done over the disease course. This could also be compared to intracellular staining of IFN-γ and IL-17 in circulating blood and/or MOG_35–55_-restimulated splenocytes at study termination.

## 5. Conclusions

RNA-Seq of brains using our mild model of EAE presented with many similar genes and pathways found in active MS lesions. This study supports the robust nature of this mild EAE model to study MS and eliminates a potential confounding factor in determining role(s) for GPCRs in EAE (and possibly MS) or involvement of GPCRs in putative therapies for MS. The major advantages of this model are that disease induction is slower to develop and results in modest clinical signs, which likely mimics human disease onset. Moreover, active immunization allows for activation of innate cells, and antigen-specific T cell and B cell responses. This model might be useful for determination of pre-clinical efficacy of novel therapies. It could even be used in direct comparison with active EAE with PTx to determine a role for IL-17 or GPCRs or establish whether differential efficacy of novel therapies exist in mild (i.e., without PTx) versus more severe (i.e., with PTx) disease.

## Figures and Tables

**Table 1:** D18 EAE Clinical Scores.

EAE/CO Mouse ID ^1^	Clinical Score
21A	2
**22A**	**0**
**23A**	**0**
**24A**	**2.5**
25A	0
**21B**	**2.25**
**22B**	**0**
23B	0
24B	2
**25B**	**2.25**

^1^ Mice were induced with EAE without PTx on day 0 then monitored over the next 18 days. Mice also received corn oil (CO) vehicle orally for the first 5 days since these mice were part of a larger study to assess the effects of a possible treatment that was delivered in CO. A and B indicate separate cohorts of mice. Brains from mice with the bold scores were submitted for RNA-Seq. Disease induction on day 18 for each cohort was as 50%.

**Table 2:** The top 20 canonical pathways: EAE symptomatic.

EAE Canonical Pathways ^1^	−log (*p*-Value)	z-Score
Pathogen-Induced Cytokine Storm Signaling Pathway	27.4	6.708
Multiple Sclerosis Signaling Pathway	21.8	5.657
Macrophage Classical Activation Signaling Pathway	21.7	4.747
Th1 Pathway	17.3	4.264
Role of Hypercytokinemia/hyperchemokinemia in the Pathogenesis of Influenza	16.8	4.359
Neuroinflammation Signaling Pathway	15.3	5
Th2 Pathway	15	2.982
Interferon Signaling	13.2	2.714
Role of Pattern Recognition Receptors in Recognition of Bacteria and Viruses	12.8	3.464
Acute Phase Response Signaling	12.4	3.051
Pyroptosis Signaling Pathway	12.4	4
IL-10 Signaling	11.9	−2.524
Crosstalk between Dendritic Cells and Natural Killer Cells	11.4	3.742
PD-1, PD-L1 cancer immunotherapy pathway	11.4	−2.5
Dendritic Cell Maturation	10.9	4.642
Natural Killer Cell Signaling	10.9	2.524
Production of Nitric Oxide and Reactive Oxygen Species in Macrophages	10.2	4
TREM1 Signaling	10.1	3.606
Phagosome Formation	9.68	5.916

^1^ The top 20 of 92 statistically significant pathways were identified in EAE Symptomatic brains. Of these 20 pathways, 18 were activated (positive z-score), and two were inhibited (negative z-score).

**Table 3:** EAE Cytokine Signaling Pathways: EAE Symptomatic.

EAE Canonical Pathways	−log (*p*-Value)	z-Score
Interferon Signaling	13.2	2.714
IL-10 Signaling	11.9	−2.524
IL-17 Signaling	5.41	3.606
IL-33 Signaling Pathway	4.71	2.714
HMGB1 Signaling	4.45	2.828
IL-6 Signaling	3.93	2.333
IL-15 Production	3.35	2.828
IL-9 Signaling	2.79	2
IL-4 Signaling	2.66	3.3
IL-8 Signaling	2.42	2.828

## Data Availability

The mouse brain EAE datasets generated and/or analyzed during the current study are available in the NCBI gene expression omnibus (GEO) GSE262437 (https://www.ncbi.nlm.nih.gov/geo/query/acc.cgi?acc=GSE262437, accessed on 13 October 2021) and were originally reported in Frodella, et al. [44]. The MS atlas was downloaded from the NCBI GEO database (https://www.ncbi.nlm.nih.gov/pmc/articles/PMC7500075/#:~:text=omnibus%20(GEO)%20database%20(-,GSE138614,-)%20as%20FASTQ%20files; accessed on 13 October 2021) as described [27].
